# Prevalence of thyroid nodules and canceration risk assessment in TIRADS, and their relationships to obesity and dysglycemia

**DOI:** 10.3389/fonc.2025.1658717

**Published:** 2025-09-29

**Authors:** Weikang Wang, Ling Xiao, Mei He, Ruimei Feng, Xiaoyin Huang, Qingling Su, Xiujian Xue, Zhijian Hu, Qian Zhang, Shanshan Du, Hong Lin, Weimin Ye

**Affiliations:** ^1^ Department of Epidemiology and Health Statistics, School of Public Health, Fujian Medical University, Fuzhou, Fujian, China; ^2^ Department of Ultrasonography, Fuqing City Hospital Affiliated to Fujian Medical University, Fuqing, Fujian, China; ^3^ Department of Epidemiology, School of Public Health, Shanxi Medical University, Taiyuan, Shanxi, China; ^4^ Department of General Surgery, Fuqing City Hospital Affiliated to Fujian Medical University, Fuqing, Fujian, China; ^5^ Department of Medical Epidemiology and Biostatistics, Karolinska Institutet, Stockholm, Sweden

**Keywords:** thyroid nodule, imaging characteristics, thyroid imaging reporting and data system, metabolic disorders, cross-sectional study

## Abstract

**Objective:**

To describe the prevalence of thyroid nodules (TNs), their ultrasonographic characteristics, and the cancer risk assessment using the Thyroid Imaging Reporting and Data System (TIRADS) in a natural population, while also exploring their association with multiple metabolic disorders. This study aims to provide insight into the disease burden of TNs in the coastal area of Southeast China.

**Design:**

A cross-sectional study.

**Setting and participants:**

A total of 6221 participants from the baseline survey of the Fuqing Cohort Study, an ongoing population-based study in a coastal city of Southeast China, were included.

**Primary and secondary outcome measures:**

TNs and its detailed ultrasonographic characteristics, risk grading in TIRADS, and metabolic factors.

**Results:**

The age- and sex-standardized prevalence of TNs was found to be 67.2%, with a higher prevalence observed in females and an increase with age. Additionally, multiple, solid, and < 10 mm TNs were common. Approximately 39.2% of participants were diagnosed with grade 3, while 2.3% were classified as grade ≥ 4a according to TIRADS. Metabolic syndrome was associated with TNs, but this association was significant only in females. The relationship between metabolic disorders and the characteristics and grade of TNs in TIRADS varied by sex.

**Conclusion:**

The prevalence of TNs in the coastal area of Southeast China is notable, with 2.3% of the population classified as grade ≥ 4a in TIRADS, indicating a high risk of cancer and necessitating further assessment for thyroid cancer. The association between TNs and metabolic disorders varies by sex and requires further confirmation.

## Introduction

Thyroid nodules (TNs), as defined by the American Thyroid Association (ATA), are discrete lesions within the thyroid gland (1), with the majority being benign, and only 5% classified as malignant (2). Similar definitions have been acknowledged by the World Health Organization (WHO) (3) and the European Thyroid Association (ETA) (4). Ultrasonography is a widely utilized tool for diagnosing TNs, where morphological characteristics such as quantity, trait, and size are integral to assessing the cancer risk according to the Thyroid Imaging Reporting and Data System (TIRADS) (5–7).

While TIRADS is not the exclusive ‘gold standard’ for TN risk stratification, it remains one of the most widely adopted and validated systems in clinical practice (2). The system categorizes TNs into five grades: grades 1–3 indicate a very low probability of malignancy, whereas grade 4a and above often signify a relatively higher risk of malignancy (8). Minor modifications to the classification systems have been implemented in various countries or regions, including the European Thyroid Association (EU-TIRADS), the Korean Society of Thyroid Radiology (K-TIRADS), and the Chinese Society of Ultrasound in Medicine and Engineering (C-TIRADS); however, the overarching principles remain consistent (9–12). Nonetheless, comprehensive descriptions of ultrasonographic morphological characteristics and their associated risk assessments in TIRADS have been less frequently reported.

The prevalence of TNs in China ranges from 36.9% in the North to 50.2% in the East (13–15), with most studies being conducted in physical examination centers (16, 17). Consequently, there is a pressing need for more screening initiatives among community residents. Fujian Province, located in the southeastern coastal region of China, is characterized by high iodine intake (18) and elevated socioeconomic levels. However, studies on the prevalence of TNs in this population are limited.

Metabolic disorders have increasingly been recognized as risk factors for TNs (19–21). The thyroid gland, which plays a crucial role in regulating metabolism, may be particularly vulnerable to changes in metabolic status. Specifically, the presence of metabolic disorders is associated with an increased risk of TN, with an odds ratio (OR) of 1.19 (95% CI: 1.11–1.28). Certain metabolic conditions, such as hypercholesterolemia (OR=1.24), high low-density lipoprotein cholesterol (OR=1.25), and hyperuricemia (OR=1.21), are independently linked to higher odds of TNs (19). However, no consistent conclusions have been reached regarding these associations (22–24). Furthermore, the specific nature of the relationship between metabolic disorders and the morphological characteristics of TNs remains undetermined.

In this context, a detailed ultrasonic thyroid screening was conducted among rural residents in a coastal city in Southeast China. The current study aims to investigate the prevalence of TNs, their ultrasonographic morphological characteristics and TIRADS grade, and to explore their associations with various metabolic disorders in the general population.

## Methods

### Study population

This cross-sectional study is part of the ongoing Fuqing Cohort Study (25), which aims to recruit over 50,000 native residents of Fuqing City, Fujian Province, located in the southeastern coastal area of China. Participants aged 35–75 years were recruited for the study. From July 2020 to June 2021, a total of 7,662 participants were enrolled from Gaoshan Town in Fuqing City. The exclusion criteria are outlined in the flowchart (**Supplementary Figure 1**). This study was conducted in accordance with the Helsinki Declaration and received approval from the ethical committee of Fujian Medical University (approval numbers: [2017-07] and [2020-58]). Prior to their participation, all study participants provided written informed consent.

### Patient and public involvement

Patients and the public were not involved in the design, conduct, reporting, or dissemination plans of this research.

### Data collection

A thyroid ultrasound examination was conducted using a color Doppler ultrasound system (Hitachi Aloka Medical, ProSound α7, Japan) by professional sonographers who were blinded to the clinical and laboratory data of the participants. During the examinations, participants were positioned supine, fully exposing their necks. The examination results, including the number of thyroid nodules, characteristics (cystic or solid), size, location, echogenicity, boundaries, and calcification, were recorded concurrently.

Following standardized protocols, an electronic questionnaire was utilized to collect demographic information, lifestyle factors (smoking, alcohol consumption, and physical activity), and medical history. Additionally, trained staff measured height, weight, waist circumference (WC), body mass index (BMI), and blood pressure (BP) levels. Fasting blood samples were analyzed to determine fasting blood glucose (FBG), 2-hour post-load blood glucose (2h PG), triglycerides (TG), high-density lipoprotein cholesterol (HDL-c), and glycosylated hemoglobin (HbA1c) levels (26).

### Definition and diagnostic criteria

The definition of a TN encompasses any area exhibiting differing echogenicity compared to the thyroid parenchyma, as established by the ATA. Solitary thyroid nodule (S-TN) is defined as the presence of a single nodule, while multiple thyroid nodules (M-TNs) refer to more than one nodule located in any region of the thyroid. The classification of TNs is based on their composition, categorized as either solid or cystic nodules. In instances where both cystic and solid nodules are identified within the same individual, they are classified as solid TNs. Furthermore, TNs are categorized based on the diameter of the largest nodule, with a cutoff of 10 mm delineating two distinct categories. The grading of TNs is evaluated according to the cumulative scores derived from the TIRADS criteria, as detailed in **Supplementary Table 1**.

Obesity and Metabolic Syndrome (MetS) are defined as follows: Obesity is classified according to body mass index (BMI), categorized into under/normal weight (< 24 kg/m²), overweight (24–28 kg/m²), and obesity (≥ 28 kg/m²). MetS is defined in accordance with the diagnostic criteria set forth by the Adult Treatment Panel III (ATP III) of the National Cholesterol Education Program (27). Participants are deemed to have MetS if three or more of the following five conditions are met: 1) Abdominal obesity (males: WC ≥ 102 cm; females: WC ≥ 88 cm); 2) Hypertriglyceridemia (TG ≥ 1.70 mmol/L); 3) Low HDL-c levels (< 1.03 mmol/L in males, or < 1.29 mmol/L in females); 4) Abnormal blood pressure (the presence of any of the following three conditions): (a) Antihypertensive treatment, (b) Systolic blood pressure (SBP) ≥ 130 mmHg, (c) Diastolic blood pressure (DBP) ≥ 85 mmHg; 5) Dysglycemia (the presence of any of the following three conditions): (a) Self-reported history of diabetes or treatment for dysglycemia, (b) Newly diagnosed diabetes according to the American Diabetes Association criteria: 2h PG ≥ 11.1 mmol/L or HbA1c ≥ 6.5%, (c) FBG ≥ 5.6 mmol/L.

### Statistical analysis

All analyses were conducted using SAS 9.4 statistical software and Microsoft Excel (Windows 10, 2016). A two-tailed p-value of < 0.05 was considered statistically significant. The Chi-squared test was employed to evaluate between-group differences in demographic characteristics, behavioral habits, and metabolism-related factors. The Wilcoxon test was utilized for analyzing skewed continuous variables. The age- and sex-standardized prevalence of TNs was calculated using the direct method based on the 2010 population distribution data in China. Logistic regression analysis was performed to compute the OR and 95% confidence intervals (CI) to establish associations between MetS and TNs. Model 1 was unadjusted; Model 2 was adjusted for age, sex, years of education, marital status, and occupation; Model 3 was additionally adjusted for smoking status and BMI. Subsequently, we examined potential multiplicative and additive interactions between MetS and various indicators. For multiplicative interactions, a cross-product term was incorporated into the regression model, with the p-value derived from the Wald test. To assess additive interactions, we employed the relative excess risk due to interaction (RERI), synergy index (S), and attributable proportion (AP) due to interaction, utilizing the spreadsheet developed by Andersson et al. (21). Finally, considering the sex-specific prevalence of MetS and TNs, we conducted a sex-stratified analysis.

### Sensitivity analysis

In the ATP III definition of MetS, abdominal obesity was defined as a WC of ≥ 102 cm and ≥ 88 cm in male and female populations, respectively, which are the cut-offs used in the United States, Canada, and Europe (28). Therefore, we redefined abdominal obesity and MetS using the cut-points recommended for the Asian population (males: WC ≥ 90 cm; females: WC ≥ 80 cm) (28, 29) and reanalyzed the associations between MetS components and TN characteristics in the multiple-variable adjusted logistic model as described above.

## Results

### Prevalence of TNs and their ultrasonographic characteristics

**Figure 1:**
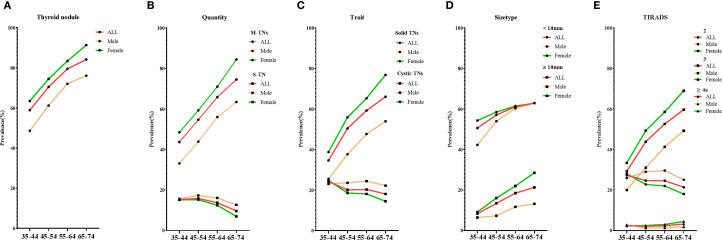
The crude prevalence of various types of TNs by age. Five line graphs labeled **(A–E)** compare the crude prevalence of various types of TNs: **(A)** for thyroid nodules, **(B)** for quantity, **(C)** for trait, **(D)** for size type, and **(E)** for TIRADS. S-TN, solitary thyroid nodule; M-TNs, multiple thyroid nodules; TIRADS, thyroid imaging reporting and data system.

A total of 6,221 participants were enrolled in this study, with 4,706 (75.7%) diagnosed with TNs, including 684 self-reported cases. The crude prevalence of TNs was 79.7% among females and 68.5% among males. Notably, the prevalence increased with age for both genders (**Figure 1A**). The age- and sex-adjusted prevalence was 67.2%, reflecting similar trends to the crude prevalence as shown in **Supplementary Table 2**.

Overall, the distribution of TNs varied significantly based on their ultrasonographic characteristics, including quantity, trait, size type, and TIRADS classification. As illustrated in **Figure 1B** (quantity), participants with M-TNs comprised 62.3% of the total population, with prevalence increasing with age, whereas those with S-TNs represented only 13.3%, with a decrease in prevalence as age increased. In terms of trait (**Figure 1C**), solid TNs exhibited a significantly higher prevalence among females compared to males and were more common in older individuals. Conversely, cystic TNs had a lower prevalence and an opposite trend. Regarding size type (**Figure 1D**), TNs ≥ 10 mm were diagnosed in only 19.9% of the female population and 10.6% of the male population. **Figure 1E** (TIRADS classification) shows that TIRADS 3 TNs were the most prevalent, accounting for 39.2% of the entire population, with prevalence increasing with age. TIRADS ≥ 4a TNs were identified in 2.3% of the population, also showing a slight increase with age. The crude and age- and sex-adjusted prevalence of TNs with these characteristics are presented in **Supplementary Table 2**.

### Sociodemographic characteristics of populations with TNs and MetS

The median age of the entire population was 57.0 years, with 36.3% being male. TNs were more prevalent among females, older individuals, those with lower education levels, widowed individuals, and participants engaged in farming or who were unemployed. Compared to non-TNs, TN cases were more likely to be non-smokers, obese, hypertensive, and hyperglycemic (**Table 1**).

**Table 1:** Distribution of sociodemographic characteristics among participants with and without TNs, and with and without MetS.

Variables	Non-TNs	TNs	*P* value^‡^	Non-MetS	MetS	*P* value^‡^
Participants	1515 (24.4)	4706 (75.7)		5204 (83.7)	1017 (16.3)	
Self-reported TNs	–	684 (11.0)		–	–	
Male	712 (47.0)	1547 (32.9)	**<0.001**	2008 (38.6)	251 (24.7)	**<0.001**
Age (years)	46 [54-62]	51 [58-66]	**<0.001**	49 [57-64]	55 [62-67]	**<0.001**
Age group (years)			**<0.001**			**<0.001**
35 - 44	333 (22.0)	476 (10.1)		751 (14.4)	58 (5.7)	
45 - 54	487 (32.1)	1160 (24.6)		1479 (28.4)	168 (16.5)	
55 - 64	429 (28.3)	1661 (35.3)		1688 (32.4)	402 (39.5)	
65 - 74	266 (17.6)	1409 (29.9)		1286 (24.7)	389 (38.2)	
Education years			**<0.001**			**<0.001**
None	327 (21.6)	1722 (36.6)		1581 (30.4)	468 (46.0)	
1 - 6	540 (35.6)	1568 (33.3)		1798 (34.6)	310 (30.5)	
7 - 9	467 (30.8)	1004 (21.3)		1309 (25.2)	162 (15.9)	
≥10	181 (11.9)	412 (8.8)		516 (9.9)	77 (7.6)	
Civil status			**<0.001**			**<0.001**
Married	1446 (95.4)	4263 (90.6)		4820 (92.6)	889 (87.4)	
Widowed	47 (3.1)	397 (8.4)		322 (6.2)	122 (12.0)	
Divorce or Single	22 (1.5)	46 (1.0)		62 (1.2)	6 (0.6)	
Occupation			**<0.001**			**<0.001**
Farmer or unemployment	950 (62.7)	3514 (74.7)		3626 (69.7)	838 (82.4)	
Worker	218 (14.4)	445 (9.5)		605 (11.6)	58 (5.7)	
Other	347 (22.9)	747 (15.9)		973 (18.7)	121 (11.9)	
PA (MET/day)	7.0 [10.0-16.6]	6.7 [10.0-16.0]	0.284	6.7 [10.0-16.6]	6.5 [9.7-14.9]	0.112
PA level			0.222			0.166
Light	377 (24.9)	1273 (27.1)		1371 (26.3)	279 (27.4)	
Moderate	658 (43.4)	2014 (42.8)		2219 (42.6)	453 (44.5)	
Vigorous	480 (31.7)	1419 (30.2)		1614 (31.0)	285 (28.0)	
Current smoking	321 (21.2)	830 (17.6)	**0.002**	1006 (19.3)	145 (14.3)	**<0.001**
BMI (kg/m^2^)	21.2 [23.3-25.7]	22.0 [24.0-26.2]		21.5 [23.4-25.4]	24.8 [26.7-29.0]	
BMI Category			**<0.001**			**<0.001**
Underweight/Normal weight	857 (56.6)	2288 (48.6)		2979 (57.2)	166 (16.3)	
Overweight	511 (33.7)	1838 (39.1)		1863 (35.8)	486 (47.8)	
Obesity	147 (9.7)	580 (12.3)		362 (7.0)	365 (35.9)	
WC (cm)	75.3 [81.5-88.6]	76.8 [83.2-89.8]	**<0.001**	75.2 [81.4-87.0]	87.0 [91.5-96.4]	**<0.001**
SBP (mmHg)	116.0 [127.5-141.5]	120.0 [132.5-148.0]	**<0.001**	117.0 [128.5-143.3]	134.0 [144.5-158.5]	**<0.001**
DBP (mmHg)	76.0 [83.5-91.0]	77.5 [84.5-92.0]	**<0.001**	76.0 [83.0-90.5]	84.0 [89.5-97.0]	**<0.001**
FBG (mmol/L)	4.6 [4.9-5.3]	4.6 [5.0-5.5]	**<0.001**	4.5 [4.9-5.3]	5.1 [5.8-7.1]	**<0.001**
TG (mmol/L)	0.80 [1.08-1.52]	0.83 [1.13-1.58]	**0.011**	0.78 [1.03-1.36]	1.51 [1.97-2.56]	**<0.001**
HDL-c (mmol/L)	1.35 [1.56-1.82]	1.35 [1.57-1.82]	0.716	1.40 [1.61-1.85]	1.16 [1.34-1.58]	**<0.001**

TNs, Thyroid nodules; MetS, Metabolic syndrome; MET, Metabolic equivalent; PA, Physical activity; BMI, Body mass index; WC, Waist circumference; SBP, Systolic blood pressure; DBP, Diastolic blood pressure; FBG, Fasting blood glucose; TG, Triglyceride; HDL-c, High-density lipoprotein cholesterol.

Data were presented as absolute numbers (percentages) for categorical variables and median [first quartile–third quartile] for skewed continuous variables.

‡P value from Chi-squared tests for categorical variables or Wilcoxon tests for skewed continuous variables between Non-TNs and TNs groups, and Non-MetS and MetS groups.

Bold P values indicate statistically significant differences.

Based on the ATP III criteria, 1,017 out of 6,221 participants were diagnosed with MetS, yielding a prevalence of 16.3% in the overall population. The sociodemographic characteristics of participants with MetS differed significantly from those without MetS, but were broadly comparable to those with TNs (**Table 1**).

### Relationship between TNs and MetS and its components

**Figure 2:**
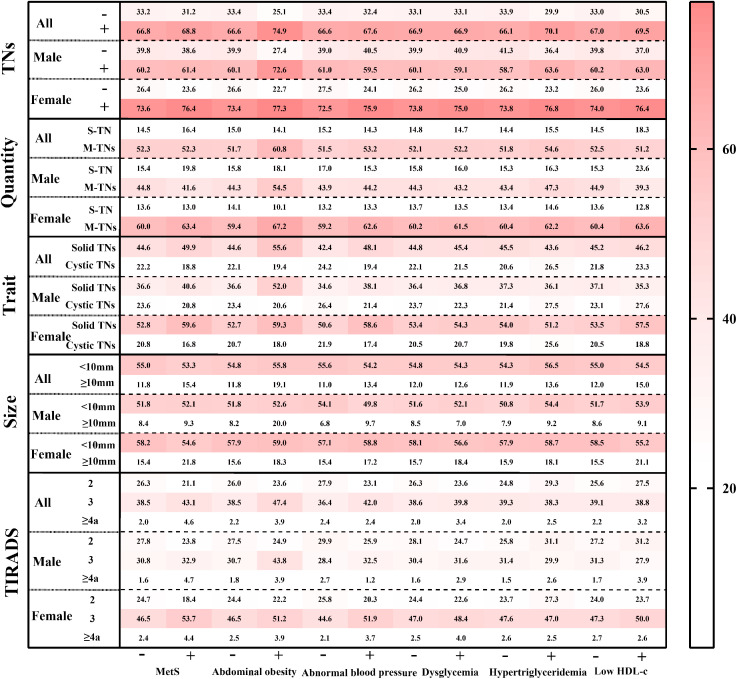
The standardized prevalence of ultrasonographic characteristics of TNs among populations with various metabolic status. TNs, thyroid nodules; S-TN, solitary thyroid nodule; M-TNs, multiple thyroid nodules; TIRADS, thyroid imaging reporting and data system. The prevalence in each grid was age- and sex- adjusted by the population distribution in China in 2010.

The crude and adjusted prevalence of TNs among populations with and without MetS and its components was calculated (see **Figure 2** and **Supplementary Table 3**). In the entire population, the prevalence of TNs was higher in MetS (68.8%) compared to those without MetS (66.8%). Similarly, participants in the positive component groups exhibited a higher prevalence of TNs than those in the negative groups: abdominal obesity (74.9% vs. 66.7%), abnormal blood pressure (67.6% vs. 66.7%), hypertriglyceridemia (70.1% vs. 66.2%), and low HDL-c (69.9% vs. 67.0%). This pattern was consistent among females. In males, however, the adjusted prevalence rates of TNs in the positive groups for hypertriglyceridemia and low HDL-c were lower than those in the negative groups (hypertriglyceridemia: 58.7% vs. 63.6%; low HDL-c: 60.2% vs. 63.0%).

In the fully adjusted logistic regression model (see **Supplementary Table 4**), MetS was significantly associated with TNs, yielding an OR of 1.25 (95% CI: 1.02–1.53) when compared to participants without MetS. Among the individual components of MetS, only abdominal obesity demonstrated a significant association, with an OR of 1.37 (95% CI: 1.08–1.73) compared to participants without abdominal obesity.

Considering the potential effects of sex, smoking status, and BMI, both multiplicative and additive interactions between MetS and each of these factors were separately tested in fully adjusted logistic regression models (see **Table 2**). Notably, only the additive interaction between MetS and sex for TNs was significant, with RERI, AP, and S recorded at 0.88 (0.02, 1.74), 0.26 (0.06, 0.46), and 1.59 (1.05, 2.42), respectively.

**Table 2:** The interaction effects between MetS and sociodemographic characteristics on TNs.

Variables	OR (95% CI)		Additive interaction			Multiplicative interaction
	Non-MetS	MetS	*RERI* (95% *CI*)	*AP* (95% *CI*)	*S* (95% *CI*)	*P* value
Sex^*^						
Male	1.00 (reference)	1.06 (0.78,1.44)	0.88 (0.02,1.74)	0.26 (0.06,0.46)	1.59 (1.05,2.42)	0.170
Female	2.43 (2.03,2.92)	3.37 (2.55,4.46)				
Current smoking^‡^						
No	1.00 (reference)	1.34 (1.07,1.67)	-0.43 (-1.10,0.24)	-0.30 (-0.86,0.26)	0.51 (0.14,1.90)	0.133
Yes	1.55 (1.29,1.87)	1.46 (0.97,2.20)				
BMI Category^†^						
Underweight/Normal weight	1.00 (reference)	1.38 (0.90,2.12)	-0.10 (-0.77,0.58)	-0.06 (-0.49,0.37)	0.86 (0.31,2.36)	0.607
Overweight/Obesity	1.31 (1.15,1.49)	1.59 (1.29,1.96)				

OR, Odds ratio; CI, Confidence intervals; MetS, Metabolic syndrome; RERI, Relative excess risk due to interaction; AP, Attributable proportion; S, Synergy index; BMI, Body mass index.

^*^OR was adjusted for age, education years, civil status, occupation, smoking status, and BMI. ^‡^OR was adjusted for age, sex, education years, civil status, occupation, and BMI. ^†^OR was adjusted for age, sex, education years, civil status, occupation, and smoking status.

### Relationships between MetS and its components and TN characteristics

**Figure 3:**
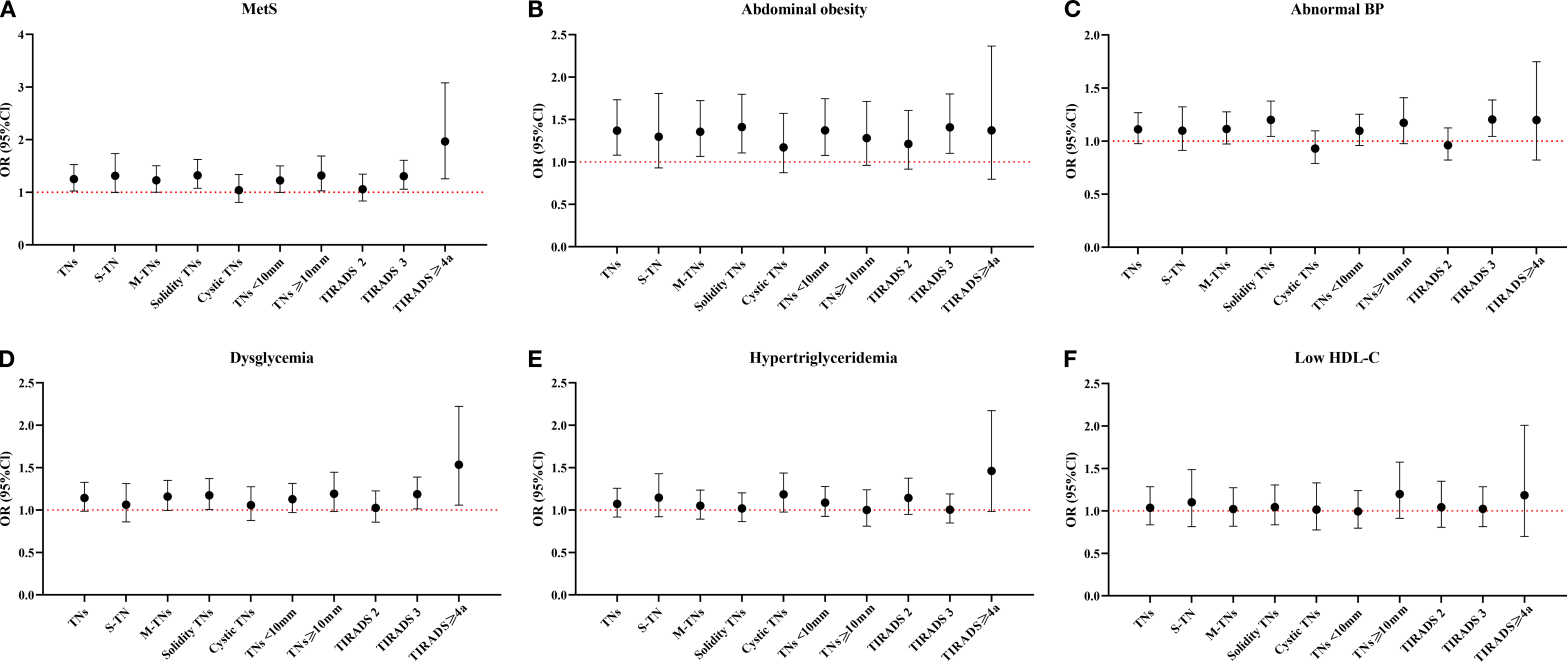
Relationship between MetS and its components and the ultrasonographic characteristics of TNs. MetS, Metabolic syndrome; TNs, Thyroid nodules; S-TN, Solitary thyroid nodule; M-TNs, Multiple thyroid nodules; TIRADS, Thyroid imaging reporting and data system; HDL-c, High density lipoprotein cholesterol. Six line graphs labeled **(A–F)** compare OR with 95%CI across MetS and its components: **(A)** for MetS, **(B)** for abdominal obesity, **(C)** for abnormal BP, **(D)** for dysglycemia, **(E)** for hypertriglyceridemia, and **(F)** for low HDL-c. The reference group of all models was Non-TN group. All models were adjusted for sex, age, education years, civil status, occupation, smoking status, and BMI.

In fully adjusted models (**Figure 3**), MetS was associated with increased risks for S-TN, M-TNs, solid TNs, TNs < 10 mm, TNs ≥ 10 mm, TIRADS 3 TNs, and TIRADS ≥ 4a TNs. Among MetS components, abdominal obesity exhibited a positive correlation with M-TNs, solid TNs, TNs < 10 mm, and TIRADS 3 TNs. Additionally, abnormal BP was significantly associated with solid TNs and TIRADS 3 TNs. Dysglycemia showed a significant association with solid TNs, TIRADS 3 TNs, and TIRADS ≥ 4a TNs, whereas hypertriglyceridemia and low HDL-c did not demonstrate significant associations with any TN ultrasonographic characteristics.

Upon stratification by sex, in males, only abdominal obesity was linked to increased risks of TNs, M-TNs, solid TNs, TNs < 10 mm, and TIRADS 3 TNs. In females, MetS was positively correlated with TNs, M-TNs, solid TNs, TNs < 10 mm, TNs ≥ 10 mm, TIRADS 3 TNs, and TIRADS ≥ 4a TNs. Abnormal BP was associated with an increased risk of solid TNs and TIRADS 3 TNs. Other MetS components—including abdominal obesity, dysglycemia, hypertriglyceridemia, and low HDL-c—did not show significant associations with any TN ultrasonographic characteristics (**Supplementary Table 5**).

### Sensitivity analysis

As demonstrated in **Supplementary Table 6**, after redefining abdominal obesity and MetS according to the criteria for the Asian population, the MetS group exhibited an increased, albeit not statistically significant, risk of total TNs, with an OR of 1.16 (95% CI: 0.98–1.36). However, when analyzing various subgroups—such as TN ultrasonographic characteristics, individual MetS components, and sex—certain associations became evident.

Among TN ultrasonographic characteristics, MetS was positively associated only with TIRADS ≥4a TNs. Regarding the individual components of MetS, abnormal BP was linked to an increased risk of solid TNs and TIRADS 3 TNs, while dysglycemia showed a significant association with solid TNs and TIRADS ≥4a TNs. The ORs for abdominal obesity and low HDL-c were not statistically significant for any ultrasonographic characteristics.

In females, MetS was positively associated with TNs, including solid TNs, TIRADS 3 TNs, and TIRADS ≥4a TNs. Abnormal BP was correlated with an increased risk of solid TNs and TIRADS 3 TNs, while hypertriglyceridemia was positively associated only with TIRADS ≥4a TNs. Notably, none of these associations were significant in males.

## Discussion

In this study, the standardized prevalence of TNs was found to be 67.2%, with a higher prevalence observed in females compared to males, and an increase noted with advancing age. Multiple, solid TNs measuring less than 10 mm were more prevalent than single, cystic TNs measuring 10 mm or greater. Approximately 39% of participants were diagnosed with TIRADS 3 TNs, while 2.3% were classified as TIRADS ≥ 4a TNs, which are considered high-risk and warrant further examination according to TIRADS guidelines. Furthermore, MetS was associated with a higher prevalence of TNs and their ultrasonographic characteristics, particularly in the female population. However, the relationship between metabolic disorders and the ultrasonographic characteristics of TNs differed between male and female populations.

In China, based on a Health Examination Cohort Study, the estimated prevalence of TNs is 31.2%, with significant regional variations ranging from 23.9% to 47.6% (30). Some screening programs among adults (aged ≥ 18 years) have reported an increasing prevalence from North to South China, with rates of 36.9% in Heilongjiang province (13), 40.1% in Beijing (14), and 50.2% in the middle and lower reaches of the Yangtze River (15). In our current study, we conducted ultrasonography screening among native villagers aged 35 to 74 years in the southeast coastal area of China. The standardized prevalence of TNs was 67.2% and demonstrated a significant increasing trend with age. The higher prevalence of TNs in our study may be partly attributed to the older age of participants and the southern geographic location. Conversely, previous studies primarily focused on populations from physical examination centers, medical institutes, or community residents examined using portable ultrasound equipment (14, 16, 17). In contrast, all participants in our study were recruited from a town, and all ultrasonic parameters were measured using two identical Color Ultrasonic Scanners equipped with a color Doppler ultrasound system. The examinations were conducted by 16 professional sonographers from a single department, all of whom had completed systematic and professional training and assessment tests and had at least 5 years of experience in ultrasound examinations. These factors contributed to a robust foundation for the diagnosis of TNs.

The ultrasonographic characteristics of TNs, including their quantity, trait, and size, are essential for risk assessment in TIRADS (31). However, the distribution of these characteristics remains poorly understood. Therefore, the present study evaluated these features during ultrasound examinations. In terms of quantity, M-TNs were found to be more prevalent than S-TN, particularly among older females, consistent with previous reports (7). Throughout the development of TNs, S-TN often progresses to M-TNs with age, while S-TN has been associated with a higher risk of thyroid cancer compared to M-TNs (32). In our population, the predominant trait of TNs was solid rather than cystic. The risk of malignancy was significantly higher in solid TNs compared to cystic ones (33). The size of TNs, especially those measuring ≥ 10 mm, is a critical factor in the clinical assessment of malignancy, fine-needle aspiration, or surgical intervention. Although the risk of cancer increases with TN size in a nonlinear manner, larger TNs exhibit a lower malignancy rate (34). In summary, the majority of TNs in the current study were multiple, solid, and < 10 mm in size, and exhibited features characteristic of benign lesions.

TIRADS provides practitioners with evidence-based recommendations for the management of TNs (35). Additionally, TIRADS grading informs decisions regarding ultrasound-guided fine-needle aspiration biopsy (FNAB), which is considered the gold standard for diagnosing thyroid cancer. Nodules graded 4a or higher are recommended for FNAB. Although FNAB is highly accurate for diagnosing thyroid malignancy, it is not suitable for population-based screening due to its invasiveness, cost, and the low prevalence of clinically significant thyroid cancer in asymptomatic individuals. Consequently, current guidelines advocate for the use of ultrasonography as the initial screening tool for TNs, reserving FNAB for nodules exhibiting suspicious sonographic features (36). TIRADS grading demonstrates high sensitivity (up to 89%) and moderate specificity (~70%), effectively reducing unnecessary FNAB for benign nodules. However, its specificity for certain histologic subtypes, such as follicular and medullary thyroid cancers, remains limited (11, 37). The European Association of Nuclear Medicine also recommends utilizing TIRADS to identify high-risk nodules for FNAB, thereby helping to reduce unnecessary procedures and enhance diagnostic specificity (38).

Despite its extensive clinical use, only a limited number of studies conducted in physical examination centers have reported on the prevalence of TNs with TIRADS 4a grade or higher, ranging from 11.0% to 24.1% in the studied populations (39, 40). In contrast, current data from natural populations indicate that TIRADS 3 TNs are the most prevalent, with a prevalence of 39.2%, while TIRADS ≥ 4a TNs were observed in only 2.3% of participants, highlighting a different distribution from that reported in physical examination centers. Therefore, further natural population-based studies with detailed descriptions of ultrasonographic characteristics are essential to provide a robust basis for evaluating the disease burden of TNs.

Additionally, we explored the association between metabolic disorders and TNs. MetS, defined by the criteria proposed by ATP III, which includes abdominal obesity for populations from the United States, Canada, and Europe, was positively associated with TNs risk in the entire population. Notably, females with MetS were more likely to be associated with TNs, whereas this association was not observed in males. The associations between MetS components and TN characteristics also varied in sex-stratified analyses. Considering the ethnic-specific definitions of MetS and abdominal obesity (28, 41), we redefined MetS and abdominal obesity as suggested for Asian populations. Similarly, an increased OR of MetS for TNs was observed in females, but not in males. Although the relationship between MetS and TNs has been investigated in a few studies (22–24), and their gender-specific associations have been reported, no consistent conclusions have been reached (42, 43). Furthermore, we investigated the association between various metabolic disorders and the quantity, trait, size, and TIRADS grade of TNs, which remain largely undisclosed. Collectively, we provided a systematic and comprehensive analysis of the relationship between metabolic factors and various TNs, emphasizing the need for further exploration of these associations to identify high-risk subpopulations for TNs, including thyroid cancer.

## Conclusions

In conclusion, TNs are prevalent in the general population of the Southeast coastal area of China, with an age-adjusted prevalence of 67.2% (95% CI: 64.6–69.8). The majority of TNs are multiple, small, solid nodules, and are diagnosed as benign lesions according to the TIRADS. TNs exhibit clear sex- and age-related trends. Additionally, metabolic disorders are associated with a higher risk of TNs, particularly among females, and the relationship between metabolic disorders and TN characteristics varies by sex.

## Data Availability

The raw data supporting the conclusions of this article will be made available by the authors, without undue reservation.
