# Supplementary material for: Prevalence of thyroid nodules and canceration risk assessment in TIRADS, and their relationships to obesity and dysglycemia
Source: Front Oncol. 2025 Sep 29;15:1658717. doi: 10.3389/fonc.2025.1658717 (PMC12515924; doi:10.3389/fonc.2025.1658717)
Supplement: Supplementary file 1 [file DataSheet1.docx]

***Supplementary materials***

| **Supplementary Table 1.** Scoring criteria for TIRADS. | | | | |
| --- | --- | --- | --- | --- |
| **Traits** | **Echo** | **Aspect ratio** | **Edge** | **Local lesion echo** |
| Cystic (0 mark) | None (0 mark) | <1 (0 mark) | Smooth (0 mark) | None (0 mark) |
| Spongy (0 mark) | High echo (1 mark) |  | Indetermination (0 mark) | Coarse calcification (1 mark) |
| Solid-cystic (1 mark) | Low echo (2 marks) | ≥1 (3 marks) | Irregular (2 marks) | Peripheral calcification (2 marks) |
| Solid (2 marks) | Super low echo (3 marks) |  | Extend beyond the thyroid (3 marks) | Punctate hyperechoic (3 marks) |

Abbreviations: TIRADS, thyroid imaging reporting and data system.

Note: The TIRADS classification level was finally defined according to clinical judgments of sonographers and the total score (0 marks: TIRADS 1; 1-2 marks: TIRADS 2; 3 marks: TIRADS 3; ≥ 4 marks: TIRADS 4a and above).

**Supplementary Table 2.** Distribution and prevalence of TNs and its ultrasonographic characteristics.

| **Variables** | **TNs** | **Quantity** | |  | **Trait** | |  | **Size** | |  | **TIRADS grade** | | |
| --- | --- | --- | --- | --- | --- | --- | --- | --- | --- | --- | --- | --- | --- |
|  |  | **S-TN** | **M-TNs** |  | **Solidity TNs** | **Cystic TNs** |  | **< 10 mm** | ≥ **10 mm** |  | **2** | **3** | ≥ **4a** |
| **N (%)** |  |  |  |  |  |  |  |  |  |  |  |  |  |
| **Total** | 4706 (75.6) | 829 (13.3) | 3877 (62.3) |  | 3454 (55.5) | 1252 (20.1) |  | 3678 (59.1) | 1028 (16.5) |  | 1496 (24.1) | 3055 (49.1) | 155 (2.5) |
| **Gender** |  |  |  |  |  |  |  |  |  |  |  |  |  |
| **Male** | 1547 (68.5) | 341 (15.1) | 1206 (53.4) |  | 1021 (45.2) | 526 (23.3) |  | 1307 (57.8) | 240 (10.6) |  | 620 (27.5) | 891 (39.4) | 36 (1.6) |
| **Female** | 3159 (79.7) | 488 (12.3) | 2671 (67.4) |  | 2433 (61.4) | 726 (18.3) |  | 2371 (59.8) | 788 (19.9) |  | 876 (22.1) | 2164 (54.6) | 119 (3.0) |
| **Age group (years)** |  |  |  |  |  |  |  |  |  |  |  |  |  |
| 35 - 44 | 476 (58.8) | 123 (15.2) | 353 (43.6) |  | 280 (34.6) | 196 (24.2) |  | 409 (50.6) | 67 (8.3) |  | 221 (27.3) | 236 (29.2) | 19 (2.4) |
| 45 - 54 | 1160 (70.4) | 260 (15.8) | 900 (54.6) |  | 830 (50.4) | 330 (20.0) |  | 940 (57.1) | 220 (13.4) |  | 405 (24.6) | 721 (43.8) | 34 (2.1) |
| 55 - 64 | 1661 (79.5) | 285 (13.6) | 1376 (65.8) |  | 1238 (59.2) | 423 (20.2) |  | 1276 (61.1) | 385 (18.4) |  | 513 (24.6) | 1099 (52.6) | 49 (2.3) |
| 65 - 74 | 1409 (84.1) | 161 (9.6) | 1248 (74.5) |  | 1106 (66) | 303 (18.1) |  | 1053 (62.9) | 356 (21.3) |  | 357 (21.3) | 999 (59.6) | 53 (3.2) |
| **Standardized Prevalence** |  |  |  |  |  |  |  |  |  |  |  |  |  |
| **Total** | 67.2 (64.6, 69.9) | 14.7 (13.4, 16.0) | 52.6 (50.3, 54.8) |  | 45.3 (43.3, 47.4) | 21.9 (20.3, 23.5) |  | 54.9 (52.5, 57.4) | 12.3 (11.3, 13.3) |  | 25.8 (24.0, 27.5) | 39.2 (37.4, 41.0) | 2.3 (1.7, 2.8) |
| **Gender** |  |  |  |  |  |  |  |  |  |  |  |  |  |
| **Male** | 60.5 (56.4, 64.7) | 15.8 (13.6, 18.1) | 44.7 (41.3, 48.2) |  | 37.0 (33.9, 40.1) | 23.5 (20.8, 26.2) |  | 52.0 (48.2, 55.8) | 8.5 (7.0, 10.0) |  | 27.6 (24.7, 30.5) | 31.1 (28.3, 33.9) | 1.9 (1.0, 2.7) |
| **Female** | 74.2 (71.0, 77.3) | 13.5 (12.1, 15.0) | 60.7 (57.9, 63.5) |  | 54.0 (51.4, 56.5) | 20.2 (18.4, 22.0) |  | 58.0 (55.2, 60.9) | 16.2 (14.8, 17.5) |  | 23.9 (22.0, 25.9) | 47.6 (45.2, 50.0) | 2.7 (2.1, 3.3) |
| **Age group (years)** |  |  |  |  |  |  |  |  |  |  |  |  |  |
| 35 - 44 | 55.9 (50.5, 61.4) | 15.3 (12.3, 18.2) | 40.7 (36.0, 45.3) |  | 31.9 (27.9, 36.0) | 24.0 (20.3, 27.6) |  | 48.2 (43.1, 53.3) | 7.7 (5.7, 9.7) |  | 27.0 (23.1, 30.9) | 26.5 (22.8, 30.1) | 2.5 (1.3, 3.7) |
| 45 - 54 | 67.7 (63.4, 71.9) | 16.2 (14.1, 18.4) | 51.4 (47.7, 55.1) |  | 46.6 (43.1, 50.0) | 21.1 (18.6, 23.6) |  | 56.1 (52.2, 60.1) | 11.5 (9.9, 13.2) |  | 25.9 (23.1, 28.7) | 39.9 (36.8, 43.1) | 1.8 (1.1, 2.5) |
| 55 - 64 | 77.6 (73.7, 81.6) | 14.3 (12.5, 16.0) | 63.4 (59.8, 66.9) |  | 56.4 (53.1, 59.7) | 21.3 (19.1, 23.4) |  | 60.9 (57.4, 64.4) | 16.8 (15.0, 18.5) |  | 25.8 (23.4, 28.1) | 49.8 (46.7, 52.9) | 2.1 (1.5, 2.7) |
| 65 - 74 | 83.7 (79.3, 88.1) | 9.8 (8.3, 11.3) | 73.9 (69.8, 78.0) |  | 65.4 (61.5, 69.2) | 18.3 (16.2, 20.4) |  | 62.9 (59.1, 66.7) | 20.8 (18.6, 23.0) |  | 21.5 (19.3, 23.8) | 59.1 (55.4, 62.7) | 3.1 (2.3, 3.9) |

Abbreviations: TNs, thyroid nodules; S-TN, solitary thyroid nodule; M-TNs, multiple thyroid nodules; TIRADS grade, the grade based on thyroid imaging reporting and data system.

Note: The standardized prevalence was adjusted by the data of the population distribution in China in 2010 and it was presented as Percentages (95% confidence intervals);

| **Supplementary Table 3.** The distribution of ultrasonographic characteristics of TNs among different populations with various metabolic status. | | | | | | | | | | | | | | | |
| --- | --- | --- | --- | --- | --- | --- | --- | --- | --- | --- | --- | --- | --- | --- | --- |
|  |  |  | | **MetS -** | **MetS +** | **Abdominal obesity -** | **Abdominal obesity +** | **Abnormal BP -** | **Abnormal BP +** | **Dysglycemia -** | **Dysglycemia +** | **Hypertriglyceridemia -** | **Hypertriglyceridemia +** | **Low HDL-c -** | **Low HDL-c +** |
| **TNs** | **All** | |  | 66.8 (64.1, 69.6) | 68.8 (60.3, 77.3) | 66.7 (64.0, 69.4) | 74.9 (61.1, 88.7) | 66.7 (62.8, 70.6) | 67.6 (63.7, 71.5) | 66.9 (64.0, 69.7) | 66.9 (60.0, 73.9) | 66.2 (63.1, 69.2) | 70.1 (63.7, 76.4) | 67.0 (64.3, 69.7) | 69.9 (60.5, 79.3) |
|  | **Male** | |  | 60.3 (56.0, 64.7) | 61.4 (48.6, 74.2) | 60.2 (56.0, 64.4) | 72.6 (47.1, 98.1) | 61.1 (54.7, 67.6) | 59.5 (54.1, 64.9) | 60.2 (55.7, 64.7) | 59.1 (48.9, 69.4) | 58.7 (53.8, 63.7) | 63.8 (56.3, 71.2) | 60.3 (56.0, 64.5) | 63.9 (47.6, 80.1) |
|  | **Female** | |  | 73.6 (70.3, 76.9) | 76.4 (65.3, 87.5) | 73.4 (70.0, 76.8) | 77.3 (68.0, 86.6) | 72.4 (68.1, 76.7) | 75.9 (70.4, 81.5) | 73.8 (70.4, 77.3) | 75.0 (65.6, 84.4) | 73.8 (70.5, 77.2) | 76.5 (66.2, 86.9) | 74.0 (70.6, 77.3) | 76.2 (67.3, 85.1) |
| **Quantity** | **All** | **S-TN** | | 14.5 (13.1, 15.9) | 16.4 (11.3, 21.6) | 15.0 (13.6, 16.4) | 14.1 (7.1, 21.2) | 15.2 (13.2, 17.1) | 14.4 (12.4, 16.3) | 14.8 (13.3, 16.2) | 14.7 (10.9, 18.6) | 14.4 (12.8, 16.0) | 15.5 (12.2, 18.8) | 14.5 (13.1, 15.9) | 18.2 (12.9, 23.5) |
|  |  | **M-TNs** | | 52.3 (49.9, 54.7) | 52.3 (45.6, 59.1) | 51.7 (49.4, 54.0) | 60.8 (48.9, 72.6) | 51.5 (48.2, 54.9) | 53.2 (49.9, 56.6) | 52.1 (49.7, 54.6) | 52.2 (46.3, 58.0) | 51.8 (49.2, 54.3) | 54.5 (49.1, 60.0) | 52.5 (50.2, 54.9) | 51.7 (44.0, 59.4) |
|  | **Male** | **S-TN** | | 15.4 (13.1, 17.7) | 19.8 (11.1, 28.4) | 15.8 (13.5, 18.1) | 18.1 (4.6, 31.6) | 17.0 (13.5, 20.4) | 15.4 (12.3, 18.4) | 15.8 (13.4, 18.3) | 16.0 (10.0, 22.0) | 15.4 (12.7, 18.1) | 16.2 (12.3, 20.2) | 15.3 (13.0, 17.6) | 23.3 (13.7, 33.0) |
|  |  | **M-TNs** | | 44.9 (41.2, 48.6) | 41.6 (32.2, 51.1) | 44.4 (40.9, 47.9) | 54.5 (32.9, 76.2) | 44.1 (38.7, 49.6) | 44.2 (39.7, 48.7) | 44.4 (40.5, 48.2) | 43.2 (34.9, 51.5) | 43.4 (39.2, 47.6) | 47.5 (41.2, 53.9) | 44.9 (41.3, 48.5) | 40.5 (27.4, 53.6) |
|  | **Female** | **S-TN** | | 13.6 (12.1, 15.1) | 13.0 (7.6, 18.4) | 14.1 (12.5, 15.6) | 10.1 (6.8, 13.4) | 13.3 (11.4, 15.1) | 13.3 (10.8, 15.8) | 13.7 (12.1, 15.2) | 13.5 (8.8, 18.2) | 13.4 (11.9, 14.9) | 14.8 (9.5, 20.1) | 13.6 (12.0, 15.1) | 12.9 (8.7, 17.0) |
|  |  | **M-TNs** | | 60.0 (57.0, 63.0) | 63.4 (53.8, 73.1) | 59.3 (56.3, 62.4) | 67.2 (58.6, 75.9) | 59.2 (55.3, 63.1) | 62.6 (57.6, 67.6) | 60.2 (57.1, 63.2) | 61.5 (53.3, 69.7) | 60.4 (57.4, 63.4) | 61.8 (52.8, 70.7) | 60.4 (57.4, 63.4) | 63.3 (55.4, 71.2) |
| **Trait** | **All** | **Solidity TNs** | | 44.6 (42.4, 46.7) | 49.9 (43.0, 56.9) | 44.6 (42.4, 46.7) | 55.5 (44.6, 66.5) | 42.4 (39.5, 45.4) | 48.2 (45.0, 51.3) | 44.8 (42.5, 47.0) | 45.4 (40.2, 50.5) | 45.5 (43.1, 47.9) | 43.6 (39.1, 48.2) | 45.2 (43.0, 47.3) | 46.1 (39.1, 53.0) |
|  |  | **Cystic TNs** | | 22.3 (20.5, 24.0) | 18.8 (14.0, 23.7) | 22.1 (20.4, 23.8) | 19.4 (11.0, 27.7) | 24.3 (21.7, 26.8) | 19.4 (17.2, 21.7) | 22.1 (20.3, 23.9) | 21.5 (16.8, 26.3) | 20.6 (18.8, 22.5) | 26.4 (22.0, 30.9) | 21.8 (20.1, 23.5) | 23.8 (17.6, 30.1) |
|  | **Male** | **Solidity TNs** | | 36.6 (33.4, 39.8) | 40.6 (30.2, 51.0) | 36.6 (33.5, 39.8) | 52.0 (31.8, 72.1) | 34.5 (29.7, 39.2) | 38.1 (34.0, 42.2) | 36.4 (33.0, 39.8) | 36.8 (29.5, 44.1) | 37.4 (33.5, 41.2) | 36.0 (30.6, 41.5) | 37.2 (34.0, 40.4) | 34.8 (23.3, 46.4) |
|  |  | **Cystic TNs** | | 23.7 (20.8, 26.6) | 20.8 (13.3, 28.3) | 23.5 (20.8, 26.3) | 20.6 (4.9, 36.3) | 26.7 (22.3, 31.0) | 21.4 (17.9, 24.9) | 23.8 (20.8, 26.8) | 22.3 (15.1, 29.5) | 21.4 (18.2, 24.6) | 27.7 (22.6, 32.9) | 23.1 (20.3, 25.9) | 29.0 (17.6, 40.5) |
|  | **Female** | **Solidity TNs** | | 52.8 (50.1, 55.6) | 59.6 (50.5, 68.8) | 52.8 (50.0, 55.6) | 59.3 (51.4, 67.1) | 50.7 (47.1, 54.2) | 58.6 (53.8, 63.4) | 53.4 (50.6, 56.3) | 54.3 (47.0, 61.5) | 54.0 (51.2, 56.8) | 51.5 (44.1, 58.8) | 53.4 (50.7, 56.2) | 57.7 (50.2, 65.2) |
|  |  | **Cystic TNs** | | 20.8 (18.9, 22.7) | 16.8 (10.6, 23.0) | 20.6 (18.7, 22.6) | 18.0 (13.0, 23.0) | 21.8 (19.3, 24.2) | 17.4 (14.5, 20.2) | 20.4 (18.5, 22.3) | 20.7 (14.7, 26.8) | 19.9 (18.0, 21.7) | 25.1 (17.7, 32.4) | 20.5 (18.6, 22.5) | 18.5 (13.7, 23.2) |
| **Size** | **All** | **<10mm** | | 55.0 (52.5, 57.5) | 53.3 (45.5, 61.1) | 54.8 (52.3, 57.3) | 55.8 (44.1, 67.4) | 55.7 (52.0, 59.3) | 54.2 (50.7, 57.7) | 54.9 (52.2, 57.5) | 54.3 (47.8, 60.9) | 54.3 (51.5, 57.1) | 56.4 (50.7, 62.2) | 55.0 (52.5, 57.5) | 54.9 (46.2, 63.6) |
|  |  | **≥10mm** | | 11.8 (10.8, 12.9) | 15.4 (12.1, 18.8) | 11.8 (10.8, 12.9) | 19.1 (11.9, 26.4) | 11.0 (9.6, 12.4) | 13.4 (11.7, 15.0) | 12.0 (10.9, 13.2) | 12.6 (10.3, 14.9) | 11.9 (10.7, 13.0) | 13.6 (10.9, 16.3) | 12.0 (10.9, 13.0) | 15.0 (11.6, 18.4) |
|  | **Male** | **<10mm** | | 52.0 (47.9, 56.0) | 52.1 (39.8, 64.4) | 51.9 (48.0, 55.9) | 52.6 (31.1, 74.1) | 54.3 (48.2, 60.4) | 49.8 (44.9, 54.7) | 51.7 (47.5, 55.9) | 52.1 (42.1, 62.2) | 50.8 (46.1, 55.5) | 54.6 (47.7, 61.5) | 51.7 (47.7, 55.7) | 54.8 (39.3, 70.3) |
|  |  | **≥10mm** | | 8.4 (6.8, 10.0) | 9.3 (5.7, 12.8) | 8.2 (6.7, 9.7) | 20.0 (6.2, 33.8) | 6.8 (4.8, 8.8) | 9.7 (7.5, 12.0) | 8.5 (6.8, 10.2) | 7.0 (5.1, 8.9) | 7.9 (6.2, 9.6) | 9.2 (6.3, 12.1) | 8.6 (7.0, 10.1) | 9.1 (4.0, 14.1) |
|  | **Female** | **<10mm** | | 58.2 (55.1, 61.2) | 54.6 (45.2, 64.0) | 57.8 (54.8, 60.9) | 59.0 (50.7, 67.4) | 57.1 (53.2, 60.9) | 58.7 (53.7, 63.8) | 58.1 (55.0, 61.2) | 56.6 (48.2, 65.0) | 57.9 (54.9, 60.9) | 58.3 (49.0, 67.6) | 58.5 (55.4, 61.5) | 55.0 (47.3, 62.6) |
|  |  | **≥10mm** | | 15.4 (14.0, 16.8) | 21.8 (16.0, 27.6) | 15.6 (14.1, 17.0) | 18.3 (14.2, 22.3) | 15.4 (13.5, 17.3) | 17.2 (14.8, 19.6) | 15.7 (14.3, 17.2) | 18.4 (14.1, 22.7) | 15.9 (14.5, 17.4) | 18.2 (13.7, 22.8) | 15.5 (14.1, 16.9) | 21.2 (16.7, 25.7) |
| **TIRADS** | **All** | **2** | | 26.3 (24.4, 28.2) | 21.1 (16.2, 26.0) | 26.0 (24.2, 27.8) | 23.6 (14.7, 32.5) | 28.0 (25.3, 30.7) | 23.1 (20.7, 25.5) | 26.3 (24.4, 28.2) | 23.6 (18.9, 28.4) | 24.8 (22.7, 26.8) | 29.1 (24.6, 33.6) | 25.6 (23.8, 27.5) | 28.0 (21.3, 34.7) |
|  |  | **3** | | 38.5 (36.6, 40.5) | 43.1 (36.8, 49.3) | 38.5 (36.6, 40.4) | 47.4 (37.4, 57.5) | 36.3 (33.7, 39.0) | 42.0 (39.1, 45.0) | 38.6 (36.5, 40.6) | 39.8 (35.1, 44.6) | 39.4 (37.2, 41.5) | 38.4 (34.0, 42.8) | 39.1 (37.2, 41.1) | 38.7 (32.6, 44.8) |
|  |  | **≥4a** | | 2.0 (1.5, 2.5) | 4.6 (1.6, 7.5) | 2.2 (1.7, 2.7) | 3.9 (0.8, 6.9) | 2.4 (1.5, 3.3) | 2.4 (1.6, 3.2) | 2.0 (1.5, 2.6) | 3.4 (1.6, 5.3) | 2.0 (1.5, 2.6) | 2.5 (1.5, 3.5) | 2.2 (1.7, 2.7) | 3.2 (0.8, 5.6) |
|  | **Male** | **2** | | 27.9 (24.8, 31.0) | 23.8 (16.0, 31.5) | 27.6 (24.7, 30.6) | 24.9 (8.3, 41.6) | 30.1 (25.4, 34.7) | 25.8 (22.0, 29.6) | 28.2 (25.0, 31.4) | 24.6 (17.3, 31.9) | 25.8 (22.3, 29.3) | 31.4 (26.0, 36.8) | 27.2 (24.2, 30.2) | 32.5 (20.4, 44.6) |
|  |  | **3** | | 30.8 (27.9, 33.7) | 32.9 (24.0, 41.8) | 30.7 (27.9, 33.5) | 43.8 (25.2, 62.3) | 28.4 (24.2, 32.6) | 32.5 (28.7, 36.3) | 30.4 (27.3, 33.5) | 31.6 (25.0, 38.2) | 31.5 (28.0, 34.9) | 29.8 (24.9, 34.7) | 31.3 (28.4, 34.2) | 27.6 (17.7, 37.4) |
|  |  | **≥4a** | | 1.6 (0.8, 2.4) | 4.7 (0.0, 9.7) | 1.8 (1.0, 2.7) | 3.9 (0.0, 9.3) | 2.7 (1.1, 4.2) | 1.2 (0.3, 2.1) | 1.6 (0.7, 2.5) | 2.9 (0.0, 5.7) | 1.5 (0.6, 2.4) | 2.6 (0.9, 4.3) | 1.7 (0.9, 2.6) | 3.8 (0.0, 8.3) |
|  | **Female** | **2** | | 24.6 (22.6, 26.7) | 18.4 (12.4, 24.4) | 24.3 (22.2, 26.4) | 22.2 (16.8, 27.7) | 25.7 (23.1, 28.4) | 20.3 (17.3, 23.3) | 24.3 (22.2, 26.4) | 22.6 (16.5, 28.7) | 23.7 (21.7, 25.7) | 26.7 (19.5, 34.0) | 24.0 (22.0, 26.1) | 23.4 (18.1, 28.7) |
|  |  | **3** | | 46.5 (44.0, 49.1) | 53.7 (44.9, 62.4) | 46.5 (43.9, 49.2) | 51.2 (44.1, 58.3) | 44.6 (41.3, 47.9) | 51.9 (47.4, 56.4) | 47.0 (44.3, 49.7) | 48.4 (41.6, 55.2) | 47.5 (45.0, 50.1) | 47.3 (39.9, 54.7) | 47.2 (44.7, 49.8) | 50.2 (43.2, 57.2) |
|  |  | **≥4a** | | 2.4 (1.8, 3.0) | 4.4 (1.3, 7.4) | 2.6 (1.9, 3.2) | 3.9 (1.4, 6.4) | 2.1 (1.4, 2.8) | 3.7 (2.3, 5.1) | 2.5 (1.9, 3.1) | 4.0 (1.6, 6.4) | 2.6 (1.9, 3.2) | 2.5 (1.5, 3.5) | 2.7 (2.0, 3.3) | 2.6 (1.1, 4.1) |

Abbreviations: TNs, thyroid nodules; S-TN, solitary thyroid nodule; M-TNs, multiple thyroid nodules; TIRADS, thyroid imaging reporting and data system.

Note: The prevalence was adjusted by the data of the population distribution in China in 2010.

| **Supplementary Table 4. The relationship between MetS and its components and TNs.** | | | | | | | |
| --- | --- | --- | --- | --- | --- | --- | --- |
| **Variables** | | **TNs** | **Non-TNs** | **Model 1** | **Model 2** | **Model 3** |  |
|  | **MetS** | 162 (10.7) | 855 (18.2) | 1.86 (1.55, 2.22) | 1.40 (1.17, 1.69) | 1.25 (1.02, 1.53) |  |
|  | **Abdominal obesity** | 141 (9.3) | 896 (19.0) | 2.29 (1.90, 2.77) | 1.53 (1.25, 1.86) | 1.37 (1.08, 1.73) |  |
|  | **Abnormal BP** | 857 (56.6) | 3060 (65.0) | 1.43 (1.27, 1.61) | 1.16 (1.02, 1.32) | 1.11 (0.97, 1.27) |  |
|  | **Dysglycemia** | 320 (21.1) | 1356 (28.8) | 1.51 (1.32, 1.74) | 1.20 (1.03, 1.38) | 1.14 (0.98, 1.33) |  |
|  | **Hypertriglyceridemia** | 291 (19.2) | 992 (21.1) | 1.12 (0.97, 1.30) | 1.18 (1.01, 1.37) | 1.07 (0.92, 1.26) |  |
|  | **Low HDL-c** | 129 (8.5) | 478 (10.2) | 1.21 (0.99, 1.49) | 1.13 (0.91, 1.39) | 1.04 (0.84, 1.29) |  |

Abbreviations: TNs, Thyroid nodules; MetS, Metabolic syndrome; BP, blood pressure; HDL-c, High-density lipoprotein cholesterol.

Note: Data was presented as odds ratio (95% confidence intervals); Model 1 was crude; Model 2 was adjusted by age, sex, education years, civil status, and occupation; Model 3 was adjusted by age, sex, education years, civil status, occupation, smoking status, and BMI.

| **Supplementary Table 5.** The relationship between MetS defined by ATP III and its components and the ultrasonographic characteristics of TNs. | | | | | | | | | | | | |
| --- | --- | --- | --- | --- | --- | --- | --- | --- | --- | --- | --- | --- |
|  | **Variables** | **TNs** | **S-TN** | **M-TNs** | **Solidity TNs** | **Cystic TNs** | **TNs <10mm** | **TNs ≥10mm** | **TIRADS 2** | **TIRADS 3** | **TIRADS ≥4a** |  |
| **All participants** | |  |  |  |  |  |  |  |  |  |  |  |
|  | **MetS** | 1.25 (1.02, 1.53) | 1.32 (1.00, 1.74) | 1.22 (1.00, 1.50) | 1.32 (1.07, 1.62) | 1.04 (0.81, 1.34) | 1.22 (1.00, 1.50) | 1.32 (1.03, 1.69) | 1.06 (0.83, 1.35) | 1.30 (1.06, 1.60) | 1.96 (1.25, 3.08) |  |
|  | **Abdominal obesity** | 1.37 (1.08, 1.73) | 1.30 (0.93, 1.81) | 1.35 (1.06, 1.72) | 1.41 (1.10, 1.80) | 1.17 (0.87, 1.58) | 1.37 (1.08, 1.74) | 1.28 (0.96, 1.71) | 1.21 (0.92, 1.61) | 1.41 (1.10, 1.80) | 1.37 (0.80, 2.37) |  |
|  | **Abnormal BP** | 1.11 (0.97, 1.27) | 1.10 (0.91, 1.32) | 1.11 (0.97, 1.27) | 1.20 (1.04, 1.38) | 0.93 (0.79, 1.09) | 1.10 (0.96, 1.25) | 1.17 (0.97, 1.41) | 0.96 (0.82, 1.12) | 1.20 (1.04, 1.39) | 1.20 (0.82, 1.75) |  |
|  | **Dysglycemia** | 1.14 (0.98, 1.33) | 1.06 (0.86, 1.31) | 1.16 (0.99, 1.35) | 1.17 (1.01, 1.37) | 1.06 (0.88, 1.27) | 1.13 (0.97, 1.31) | 1.19 (0.98, 1.45) | 1.02 (0.86, 1.23) | 1.19 (1.01, 1.39) | 1.53 (1.06, 2.22) |  |
|  | **Hypertriglyceridemia** | 1.07 (0.92, 1.26) | 1.15 (0.92, 1.43) | 1.05 (0.89, 1.24) | 1.02 (0.86, 1.20) | 1.18 (0.98, 1.44) | 1.09 (0.93, 1.28) | 1.00 (0.81, 1.24) | 1.14 (0.95, 1.38) | 1.00 (0.85, 1.19) | 1.46 (0.98, 2.17) |  |
|  | **Low HDL-c** | 1.04 (0.84, 1.29) | 1.10 (0.82, 1.49) | 1.02 (0.82, 1.28) | 1.05 (0.84, 1.31) | 1.01 (0.78, 1.33) | 1.00 (0.80, 1.24) | 1.20 (0.91, 1.58) | 1.05 (0.81, 1.35) | 1.03 (0.82, 1.29) | 1.19 (0.70, 2.01) |  |
| **Male** | |  |  |  |  |  |  |  |  |  |  |  |
|  | **MetS** | 1.05 (0.77, 1.44) | 1.21 (0.78, 1.88) | 1.01 (0.72, 1.40) | 1.08 (0.77, 1.51) | 1.00 (0.68, 1.49) | 1.05 (0.76, 1.45) | 1.06 (0.65, 1.73) | 1.05 (0.72, 1.53) | 1.02 (0.72, 1.44) | 1.85 (0.71, 4.80) |  |
|  | **Abdominal obesity** | 2.05 (1.04, 4.03) | 2.26 (0.89, 5.70) | 2.00 (1.00, 4.00) | 2.69 (1.33, 5.45) | 1.08 (0.45, 2.58) | 2.04 (1.02, 4.08) | 2.07 (0.83, 5.17) | 1.35 (0.60, 3.06) | 2.53 (1.23, 5.20) | 3.10 (0.65, 14.74) |  |
|  | **Abnormal BP** | 1.04 (0.85, 1.27) | 0.91 (0.69, 1.21) | 1.09 (0.88, 1.35) | 1.16 (0.93, 1.45) | 0.86 (0.67, 1.10) | 1.00 (0.81, 1.22) | 1.34 (0.94, 1.91) | 0.92 (0.73, 1.17) | 1.17 (0.93, 1.48) | 0.59 (0.29, 1.23) |  |
|  | **Dysglycemia** | 1.16 (0.93, 1.44) | 1.05 (0.77, 1.44) | 1.19 (0.95, 1.49) | 1.21 (0.96, 1.52) | 1.07 (0.81, 1.40) | 1.15 (0.92, 1.43) | 1.23 (0.88, 1.73) | 1.05 (0.81, 1.37) | 1.21 (0.96, 1.54) | 1.93 (0.94, 3.95) |  |
|  | **Hypertriglyceridemia** | 1.01 (0.81, 1.27) | 1.07 (0.78, 1.48) | 0.99 (0.78, 1.26) | 0.91 (0.71, 1.16) | 1.22 (0.93, 1.61) | 1.05 (0.83, 1.32) | 0.80 (0.55, 1.18) | 1.19 (0.91, 1.55) | 0.87 (0.68, 1.13) | 1.33 (0.61, 2.93) |  |
|  | **Low HDL-c** | 0.83 (0.57, 1.22) | 1.41 (0.87, 2.31) | 0.67 (0.45, 1.02) | 0.74 (0.49, 1.14) | 1.00 (0.63, 1.59) | 0.82 (0.56, 1.22) | 0.88 (0.47, 1.63) | 0.99 (0.64, 1.54) | 0.70 (0.45, 1.09) | 1.18 (0.34, 4.10) |  |
| **Female** | |  |  |  |  |  |  |  |  |  |  |  |
|  | **MetS** | 1.37 (1.05, 1.79) | 1.43 (0.99, 2.05) | 1.36 (1.04, 1.77) | 1.45 (1.11, 1.89) | 1.10 (0.79, 1.53) | 1.34 (1.03, 1.76) | 1.44 (1.06, 1.95) | 1.09 (0.79, 1.49) | 1.45 (1.11, 1.90) | 2.04 (1.21, 3.42) |  |
|  | **Abdominal obesity** | 1.27 (0.97, 1.67) | 1.24 (0.84, 1.82) | 1.27 (0.96, 1.67) | 1.27 (0.96, 1.69) | 1.23 (0.88, 1.73) | 1.30 (0.98, 1.72) | 1.18 (0.85, 1.64) | 1.19 (0.86, 1.65) | 1.30 (0.98, 1.72) | 1.23 (0.68, 2.24) |  |
|  | **Abnormal BP** | 1.15 (0.97, 1.37) | 1.25 (0.98, 1.60) | 1.13 (0.95, 1.35) | 1.22 (1.02, 1.45) | 0.99 (0.79, 1.23) | 1.16 (0.97, 1.38) | 1.14 (0.91, 1.42) | 0.99 (0.80, 1.22) | 1.22 (1.02, 1.47) | 1.47 (0.94, 2.29) |  |
|  | **Dysglycemia** | 1.12 (0.92, 1.38) | 1.08 (0.81, 1.44) | 1.13 (0.92, 1.39) | 1.15 (0.93, 1.41) | 1.05 (0.81, 1.36) | 1.11 (0.90, 1.37) | 1.16 (0.91, 1.49) | 1.00 (0.78, 1.28) | 1.16 (0.94, 1.44) | 1.40 (0.90, 2.17) |  |
|  | **Hypertriglyceridemia** | 1.11 (0.89, 1.39) | 1.24 (0.91, 1.70) | 1.09 (0.86, 1.37) | 1.09 (0.86, 1.38) | 1.19 (0.90, 1.57) | 1.12 (0.89, 1.41) | 1.08 (0.82, 1.42) | 1.13 (0.86, 1.48) | 1.09 (0.86, 1.38) | 1.48 (0.92, 2.39) |  |
|  | **Low HDL-c** | 1.15 (0.89, 1.50) | 0.99 (0.68, 1.45) | 1.19 (0.91, 1.55) | 1.19 (0.91, 1.56) | 1.04 (0.75, 1.45) | 1.09 (0.84, 1.43) | 1.35 (0.98, 1.85) | 1.09 (0.79, 1.49) | 1.17 (0.89, 1.54) | 1.29 (0.71, 2.32) |  |

Abbreviations: MetS, Metabolic syndrome; BP, blood pressure; TNs, thyroid nodules; S-TN, solitary thyroid nodule; M-TNs, multiple thyroid nodules; TIRADS, thyroid imaging reporting and data system; HDL-c, high-density lipoprotein cholesterol.

Note: Data were presented as odds ratio (95% confidence intervals); The reference group of all models was Non-TN group; All models were adjusted for age, education years, civil status, occupation, smoking status, and BMI.

| **Supplementary Table 6.** The relationship between MetS defined by revised ATP III and its components and the ultrasonographic characteristics of TNs. | | | | | | | | | | | | |
| --- | --- | --- | --- | --- | --- | --- | --- | --- | --- | --- | --- | --- |
|  | **Variables** | **TNs** | **S-TN** | **M-TNs** | **Solidity TNs** | **Cystic TNs** | **TNs <10mm** | **TNs ≥10mm** | **TIRADS 2** | **TIRADS 3** | **TIRADS ≥4a** |  |
| **All participants** | |  |  |  |  |  |  |  |  |  |  |  |
|  | **MetS** | 1.16 (0.98, 1.36) | 1.19 (0.95, 1.50) | 1.14 (0.96, 1.35) | 1.17 (0.99, 1.39) | 1.10 (0.89, 1.35) | 1.16 (0.98, 1.37) | 1.14 (0.92, 1.41) | 1.09 (0.90, 1.33) | 1.15 (0.97, 1.37) | 1.87 (1.25, 2.78) |  |
|  | **Abdominal obesity** | 0.98 (0.84, 1.15) | 0.92 (0.73, 1.15) | 0.99 (0.84, 1.17) | 0.99 (0.84, 1.18) | 0.93 (0.76, 1.14) | 0.97 (0.83, 1.14) | 1.01 (0.81, 1.26) | 0.91 (0.75, 1.11) | 1.02 (0.86, 1.21) | 0.86 (0.55, 1.35) |  |
|  | **Abnormal BP** | 1.11 (0.98, 1.27) | 1.11 (0.92, 1.33) | 1.11 (0.97, 1.27) | 1.20 (1.04, 1.37) | 0.94 (0.79, 1.10) | 1.10 (0.96, 1.25) | 1.17 (0.98, 1.41) | 0.97 (0.83, 1.13) | 1.20 (1.04, 1.38) | 1.17 (0.80, 1.71) |  |
|  | **Dysglycemia** | 1.15 (0.98, 1.35) | 1.08 (0.86, 1.36) | 1.16 (0.99, 1.37) | 1.18 (1.00, 1.40) | 1.06 (0.87, 1.30) | 1.13 (0.96, 1.34) | 1.22 (0.99, 1.50) | 1.06 (0.87, 1.29) | 1.18 (0.99, 1.40) | 1.58 (1.07, 2.33) |  |
|  | **Hypertriglyceridemia** | 1.09 (0.94, 1.28) | 1.16 (0.93, 1.44) | 1.07 (0.91, 1.26) | 1.04 (0.89, 1.23) | 1.19 (0.98, 1.44) | 1.11 (0.95, 1.30) | 1.01 (0.81, 1.24) | 1.15 (0.95, 1.38) | 1.03 (0.87, 1.22) | 1.61 (1.09, 2.36) |  |
|  | **Low HDL-c** | 1.06 (0.87, 1.29) | 1.13 (0.86, 1.50) | 1.04 (0.85, 1.28) | 1.07 (0.87, 1.32) | 1.02 (0.79, 1.31) | 1.02 (0.83, 1.25) | 1.21 (0.93, 1.56) | 1.06 (0.83, 1.35) | 1.05 (0.84, 1.29) | 1.19 (0.72, 1.95) |  |
| **Male** | |  |  |  |  |  |  |  |  |  |  |  |
|  | **MetS** | 1.03 (0.80, 1.32) | 1.15 (0.80, 1.64) | 0.99 (0.76, 1.29) | 1.03 (0.79, 1.35) | 1.03 (0.75, 1.41) | 1.04 (0.81, 1.35) | 0.94 (0.63, 1.41) | 1.09 (0.81, 1.47) | 0.95 (0.72, 1.25) | 2.22 (0.96, 5.10) |  |
|  | **Abdominal obesity** | 0.91 (0.71, 1.17) | 0.95 (0.66, 1.36) | 0.90 (0.69, 1.17) | 0.90 (0.69, 1.18) | 0.93 (0.68, 1.28) | 0.88 (0.68, 1.14) | 1.14 (0.76, 1.71) | 0.91 (0.68, 1.24) | 0.90 (0.68, 1.19) | 1.18 (0.46, 3.08) |  |
|  | **Abnormal BP** | 1.03 (0.84, 1.27) | 0.92 (0.69, 1.22) | 1.08 (0.87, 1.33) | 1.15 (0.92, 1.44) | 0.87 (0.67, 1.11) | 1.00 (0.81, 1.23) | 1.29 (0.91, 1.82) | 0.93 (0.73, 1.18) | 1.16 (0.92, 1.46) | 0.60 (0.29, 1.24) |  |
|  | **Dysglycemia** | 1.13 (0.89, 1.43) | 1.02 (0.72, 1.43) | 1.16 (0.91, 1.48) | 1.16 (0.91, 1.49) | 1.07 (0.80, 1.43) | 1.12 (0.88, 1.42) | 1.18 (0.82, 1.69) | 1.09 (0.82, 1.44) | 1.13 (0.88, 1.46) | 1.99 (0.95, 4.20) |  |
|  | **Hypertriglyceridemia** | 1.07 (0.85, 1.33) | 1.13 (0.82, 1.55) | 1.05 (0.83, 1.32) | 0.96 (0.75, 1.22) | 1.27 (0.97, 1.68) | 1.11 (0.88, 1.40) | 0.83 (0.56, 1.21) | 1.24 (0.95, 1.61) | 0.93 (0.72, 1.20) | 1.31 (0.59, 2.87) |  |
|  | **Low HDL-c** | 0.95 (0.68, 1.34) | 1.51 (0.97, 2.33) | 0.80 (0.56, 1.15) | 0.88 (0.61, 1.28) | 1.09 (0.72, 1.64) | 0.92 (0.64, 1.30) | 1.17 (0.70, 1.96) | 1.12 (0.76, 1.66) | 0.83 (0.57, 1.22) | 0.94 (0.27, 3.22) |  |
| **Female** | |  |  |  |  |  |  |  |  |  |  |  |
|  | **MetS** | 1.25 (1.00, 1.56) | 1.26 (0.92, 1.72) | 1.24 (0.99, 1.56) | 1.27 (1.01, 1.59) | 1.17 (0.88, 1.55) | 1.25 (0.99, 1.57) | 1.23 (0.94, 1.61) | 1.11 (0.85, 1.46) | 1.27 (1.01, 1.60) | 1.79 (1.12, 2.86) |  |
|  | **Abdominal obesity** | 0.99 (0.80, 1.22) | 0.90 (0.67, 1.22) | 1.01 (0.82, 1.26) | 1.02 (0.82, 1.27) | 0.92 (0.70, 1.19) | 1.00 (0.81, 1.24) | 0.96 (0.73, 1.26) | 0.89 (0.69, 1.14) | 1.06 (0.85, 1.32) | 0.72 (0.43, 1.22) |  |
|  | **Abnormal BP** | 1.15 (0.97, 1.37) | 1.27 (0.99, 1.62) | 1.13 (0.95, 1.35) | 1.22 (1.02, 1.46) | 1.00 (0.80, 1.24) | 1.16 (0.97, 1.38) | 1.15 (0.92, 1.44) | 1.00 (0.81, 1.23) | 1.23 (1.02, 1.47) | 1.42 (0.91, 2.22) |  |
|  | **Dysglycemia** | 1.17 (0.93, 1.47) | 1.16 (0.84, 1.59) | 1.17 (0.93, 1.47) | 1.20 (0.95, 1.51) | 1.07 (0.80, 1.42) | 1.15 (0.91, 1.44) | 1.24 (0.95, 1.63) | 1.04 (0.79, 1.37) | 1.21 (0.96, 1.52) | 1.46 (0.91, 2.34) |  |
|  | **Hypertriglyceridemia** | 1.10 (0.88, 1.37) | 1.21 (0.89, 1.65) | 1.08 (0.86, 1.35) | 1.08 (0.86, 1.36) | 1.15 (0.87, 1.52) | 1.11 (0.88, 1.40) | 1.06 (0.81, 1.39) | 1.09 (0.83, 1.42) | 1.08 (0.85, 1.36) | 1.66 (1.04, 2.63) |  |
|  | **Low HDL-c** | 1.12 (0.87, 1.43) | 0.97 (0.68, 1.39) | 1.15 (0.89, 1.48) | 1.15 (0.89, 1.49) | 1.00 (0.72, 1.37) | 1.07 (0.83, 1.39) | 1.25 (0.93, 1.69) | 1.04 (0.77, 1.40) | 1.14 (0.88, 1.48) | 1.30 (0.75, 2.26) |  |

Abbreviations: MetS, Metabolic syndrome; BP, blood pressure; TNs, thyroid nodules; S-TN, solitary thyroid nodule; M-TNs, multiple thyroid nodules; TIRADS, thyroid imaging reporting and data system; HDL-c, high-density lipoprotein cholesterol.

Note: Data were presented as odds ratio (95% confidence intervals); The reference group of all models was Non-TN group; All models were adjusted for age, education years, civil status, occupation, smoking status, and BMI.


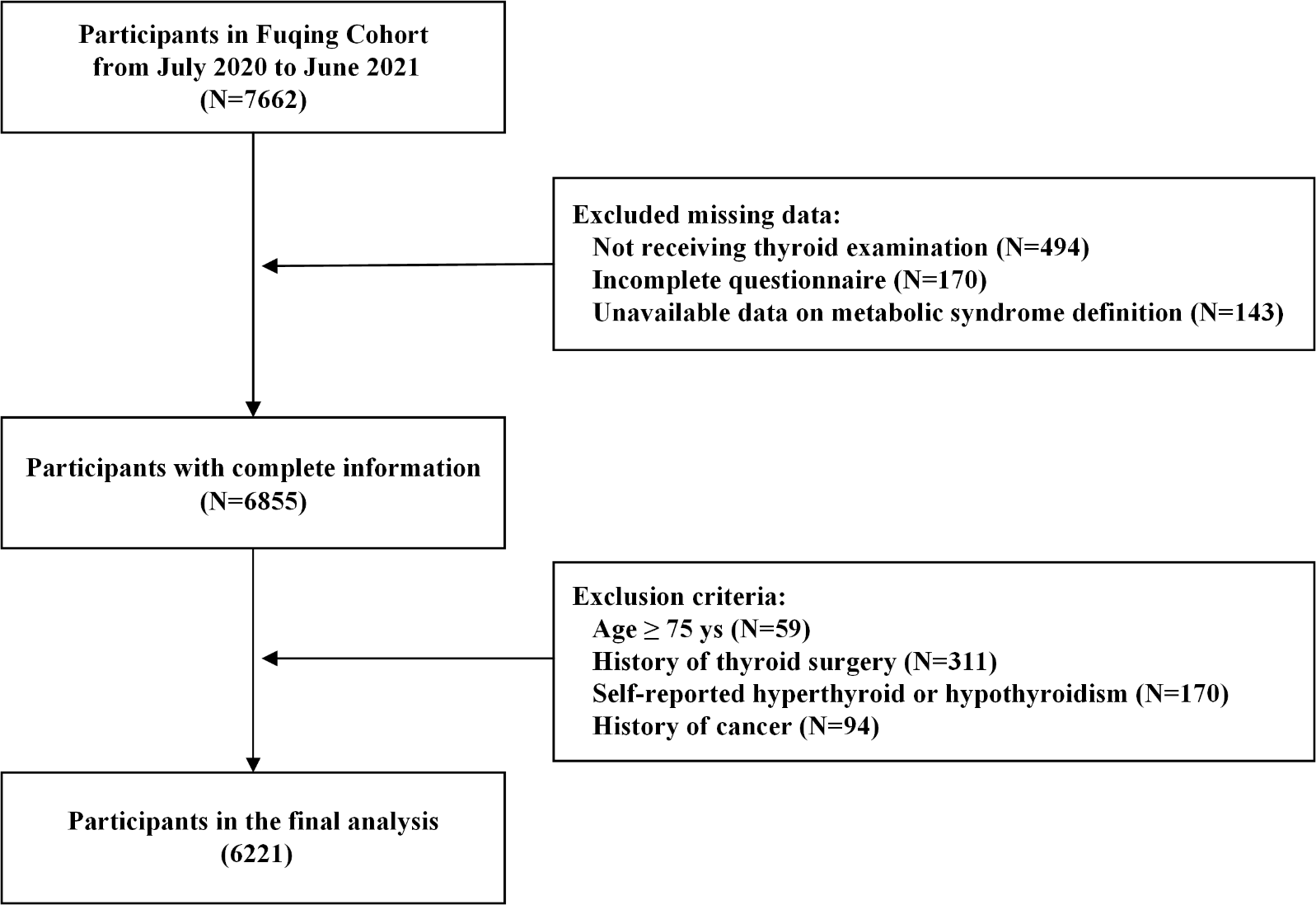


**Supplementary Figure 1.** Flowchart of inclusion of study participants.
